# Rare Exonic Minisatellite Alleles in *MUC2* Influence Susceptibility to Gastric Carcinoma

**DOI:** 10.1371/journal.pone.0001163

**Published:** 2007-11-14

**Authors:** Yun Hee Jeong, Min Chan Kim, Eun-Kyung Ahn, So-Young Seol, Eun-Ju Do, Hong-Jo Choi, In-Sun Chu, Wun-Jae Kim, Woo Jin Kim, Yangil Sunwoo, Sun-Hee Leem

**Affiliations:** 1 Department of Biological Science, Dong-A University, Busan, Korea; 2 Department of Surgery, College of Medicine, Dong-A University, Busan, Korea; 3 Medical Genomics Research Center, Korean Research Institute of Bioscience and Biotechnology (KRIBB), Daejeon, Korea; 4 Department of Urology, College of Medicine, Chungbuk National University, Cheongju, Korea; 5 Department of Internal Medicine, College of Medicine, Kangwon National University, Chuncheon, Kangwon-Do, Korea; University of Bern, Switzerland

## Abstract

**Background:**

Mucins are the major components of mucus and their genes share a common, centrally-located region of sequence that encodes tandem repeats. Mucins are well known genes with respect to their specific expression levels; however, their genomic levels are unclear because of complex genomic properties. In this study, we identified eight novel minisatellites from the entire *MUC2* region and investigated how allelic variation in these minisatellites may affect susceptibility to gastrointestinal cancer.

**Methodology/Principle Findings:**

We analyzed genomic DNA from the blood of normal healthy individuals and multi-generational family groups. Six of the eight minisatellites exhibited polymorphism and were transmitted meiotically in seven families, following Mendelian inheritance. Furthermore, a case-control study was performed that compared genomic DNA from 457 cancer-free controls with DNA from individuals with gastric (455), colon (192) and rectal (271) cancers. A statistically significant association was identified between rare exonic *MUC2*-MS6 alleles and the occurrence of gastric cancer: odds ratio (OR), 2.56; 95% confidence interval (CI), 1.31–5.04; and *p = *0.0047. We focused on an association between rare alleles and gastric cancer. Rare alleles were divided into short (40, 43 and 44) and long (47, 50 and 54), according to their TR (tandem repeats) lengths. Interestingly, short rare alleles were associated with gastric cancer (OR = 5.6, 95% CI: 1.93–16.42; *p = *0.00036). Moreover, hypervariable *MUC2* minisatellites were analyzed in matched blood and cancer tissue from 28 patients with gastric cancer and in 4 cases of *MUC2-*MS2, minisatellites were found to have undergone rearrangement.

**Conclusions/Significance:**

Our observations suggest that the short rare *MUC2*-MS6 alleles could function as identifiers for risk of gastric cancer. Additionally, we suggest that minisatellite instability might be associated with *MUC2* function in cancer cells.

## Introduction

Mucins are high molecular weight epithelial glycoproteins that are contained in mucus, a viscous secretion that covers epithelial surfaces [1]. Mucin oligosaccharides are attached to the protein backbone via O-glycosidic linkages to the hydroxyl groups of serine and threonine. They play an important role in the protection of epithelial cells and have been implicated in the process of epithelial renewal and differentiation [2], [3]. Twenty human mucin genes have been identified and classified functionally. They comprise both secreted gel-forming mucins and transmembrane mucins, although some *MUC* gene products do not fit well into either class [1]. Four of these *MU*C genes (*MUC6*, *MUC2, MUC5AC* and *MUC5B*) are believed to encode gel-forming mucins and they are clustered between *H-ras* and *IGF2* on chromosome 11p15.5 [4].

Analysis of human *MUC* gene sequences has identified the presence of several features that may represent important functional domains in mucin glycoproteins [1]. In the central region of each mucin, there are a variable number of tandem repeats (VNTR) comprised of threonine-, serine- and proline-rich repeat peptides [1]. These tandem repeat (TR) units are characteristic of mucin glycoproteins and exhibit substantial genetic variation with respect to TR number. Although its effect on function remains unclear, the TR number of several genes (*H-ras, hTERT*, mucins and insulin genes) has been associated with susceptibility to certain human diseases [5]–[12]. Individuals with small *MUC1* genotypes (SS) are at increased odds (4.3; 1.8–10.5) for gastric carcinoma development [7]. There are several lines of evidence which indicate that VNTR polymorphisms affect gene expression [13], [14]. These include problems arising from high levels of polymorphism and consequent heterozygosity, which influence both the haplotypes present in a given cell, as well as which haplotype is responsible for gene expression. Comparison of normal and cancer tissue has revealed rearrangement of at least two VNTRs in *hTERT* and has suggested that minisatellites might be associated with activation of telomerase expression in cancer cells [15].

Human minisatellites are highly variable TR sequences located predominantly in subtelomeric regions of the chromosome [16], [17]. *MUC2* is located in the subtelomeric region of chromosome 11 and its cDNA is 15,563 bp in length [18]. The TR domain (*MUC2-*MS7 region in this study) is polymorphic due to allelic variations in the length of *MUC2* mRNA [9], [19]. In contrast to *MUC2*, *MUC5B* does not exhibit allelic variation and the number of TRs encoded does not vary [20]. However, a polymorphic locus was identified in intron 36 of *MUC5B* [21]. Thus, substantial allelic differences in the length of mucins may occur in both exonic and intronic regions, and these may have functional consequences that lead to differences in disease susceptibility.

*MUC2* is the major gel forming mucin secreted by goblet cells of the intestine and it is the main structural component of the mucus gel [22]. Expression of *MUC2* is reduced in colorectal adenocarcinoma, although expression is observed in mucinous carcinomas [23]. Normal gastric mucosa show little or no expression of *MUC2*, but gastric carcinoma mucosa exhibit a heterogeneous mucin expression pattern. Mucin genes expressed in normal gastric mucosa include *MUC1*, *MUC5AC* and *MUC6* [24], [25], whereas *MUC2* is expressed aberrantly in 30% of gastric carcinomas [25].

The first aim of this study was to determine the genomic features of the *MUC2* region. Characterization of its structure indicated the presence of repeated regions within *MUC2* and both exonic and intronic minisatellite regions were identified. In addition, we examined the multiallelic properties of these analyzed minisatellites. Since specific alleles of polymorphic minisatellites are often linked to elevated odds for disease [5]–[12], our second aim was to identify links between certain specific alleles of *MUC2* minisatellites and susceptibility for gastrointestinal cancer. We compared the allelic distribution in DNA samples from cancer-free controls and patients with gastrointestinal cancer. Furthermore, we compared the number of VNTR repeats in DNA from matched blood and cancer tissue obtained from 28 gastric cancer patients. Here, we report that allelic variations in the minisatellites of *MUC2* are related to susceptibility to gastric cancer.

## Results

### Analysis of minisatellite polymorphisms in *MUC2*

**Figure 1:**
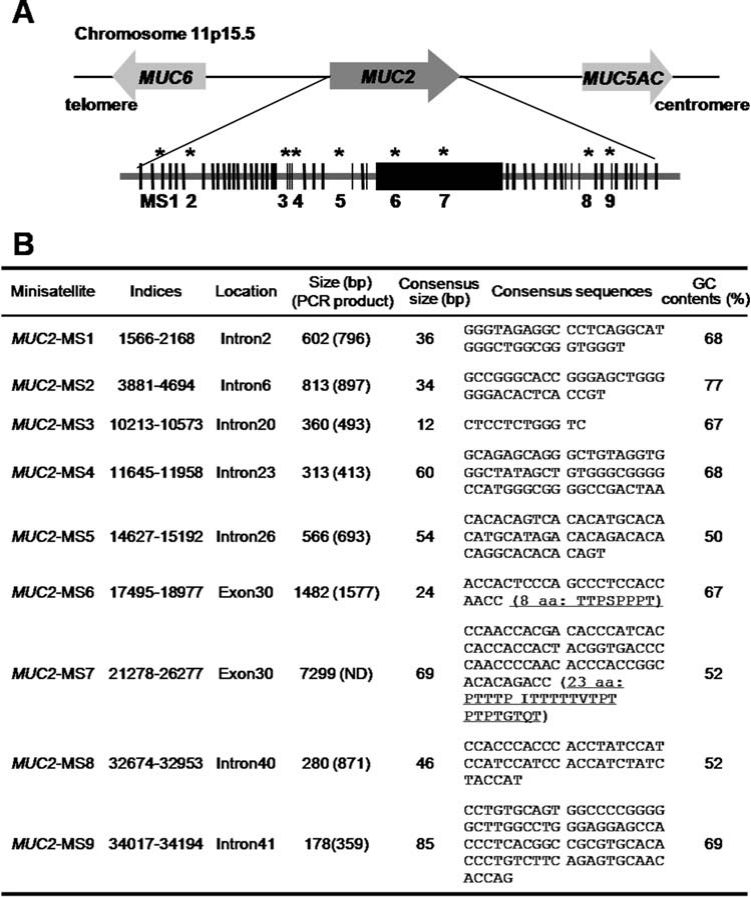
Minisatellites in *MUC2*. A. Structure of the genomic region around *MUC2*. It is predicted that 49 exons (black boxes) encode *MUC2*. Approximate positions of minisatellites identified by the Tandem Repeats Finder Program [22], are indicated by asterisks and numbers (MS1, 2, 3, 4, 5, 6, 7, 8, 9). B. The sequences of nine minisatellite repeat units. Positions of indices (1–37,982 bp) were determined using genomic information from the UCL site [21].

Analysis of the *MUC2* genomic DNA sequence (1–37,982 bp) obtained from the UCL homepage indicated the presence of 49 exons and 48 introns (Figure 1A) [26]. Furthermore, analysis with the Tandem Repeats Finder program [27] identified 9 tandem repeats (algorithm scores >300). The length of these repeats, their locations and consensus sequences are presented in Figure 1B. Given their presence in exons, two minisatellites (*MUC2-*MS6 and *-*MS7) also contained repeated amino acids sequences (Figure 1B). The degree of polymorphism within the minisatellites was examined by PCR using diagnostic primers against human genomic DNA samples isolated from cancer-free control individuals.

**Figure 2:**
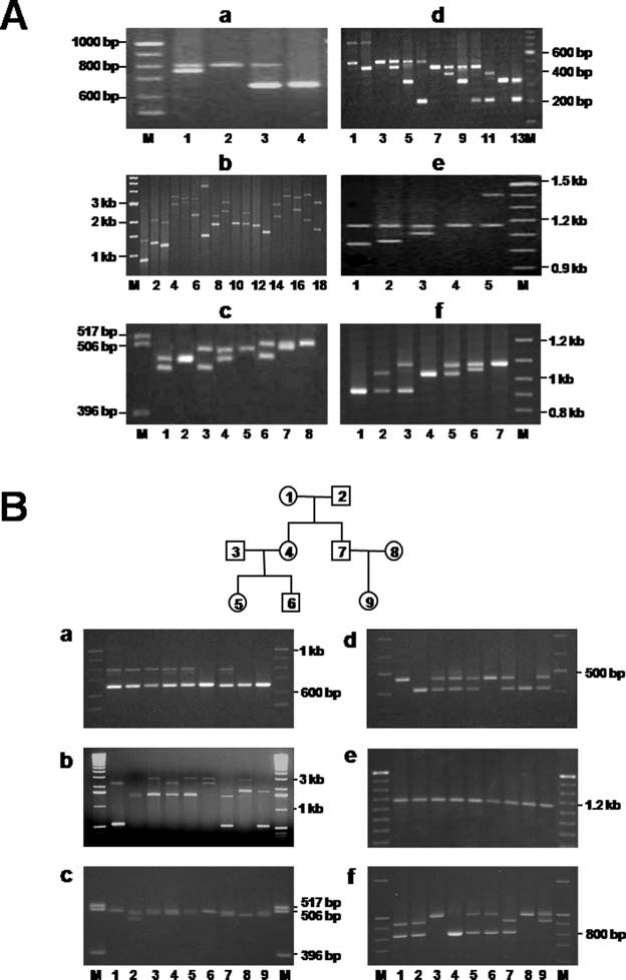
Polymorphic patterns of *MUC2* minisatellites (MS1, 2, 3, 4, 6, and 8) and their Inheritance. A. Polymorphic patterns of MS1 (a), MS2 (b), MS3 (c), MS4 (d), MS6 (e), and MS8 (f). Minisatellites were PCR-amplified from genomic DNA of control samples using diagnostic primers. Allele frequency, size of PCR products and repeat number are indicated in Table S1. Haplotype patterns are numbered according to each minisatellite. Size markers (M) are given in kb (1 kb size marker) or bp (100 bp size marker). B. Meiotic inheritance of *MUC2* minisatellites in a three-generation family: MS1 (a), MS2 (b), MS3 (c), MS4 (d), MS6 (e), and MS8 (f). PCR primers specific to *MUC2* minisatellites were used to analyze minisatellite length in genomic DNA from family members. The pedigree demonstrates the relationship between family groups used in this study: first generation (lanes 1 and 2, grandfather and grandmother, respectively); second generation (lanes 3 and 7, fathers; lanes 4 and 8, mothers); and third generation (lanes 5 and 6, children from parents 3 and 4; lane 9, child from parents 7 and 8). M corresponds to the size marker.

We found seven intronic and two exonic *MUC2* minisatellites and their allelic size and frequencies are presented in Figure 1. *MUC2-*MS1 was found in intron 2 and exhibited a polymorphic pattern in cancer-free controls. The three alleles identified in MS1 ranged between 664 and 796 bp in length and contained 12–15 copies of the repeat unit (Table S1 and Figure 2A *–*a). In intron 6, 68 alleles of *MUC2-*MS2 were identified in DNA from 100 control samples (number of alleles: N = 200) using specific primers from minisatellite-flanking sequences (Table S1 and Figure 2A–b). Most individuals were heterozygous for *MUC2-*MS2 and the number of repeats varied from 9 to 115 with 86 repeats present in the most common allele (6% frequency). In intron 20, *MUC2-*MS3 was found to be polymorphic and the number of repeats varied from 25.5 to 28, with the latter present in the most common allele (44%; Table S1 and Figure 2A–c). In intron 23, *MUC2-*MS4 was found to contain 4 alleles in which the number of repeats ranged between 1 and 10, with the most common allele (56%) containing 5 repeats (Table S1 and Figure 2A–d). In intron 40, four alleles were identified in *MUC2-*MS8 from 670 cases control samples (Table S1 and Figure 2A–f). The number of repeats varied between 6 and 10, with the latter contained in the most common allele (57%). No polymorphisms were observed in either *MUC2-*MS5 or *MUC2-*MS9, which were found in introns 26 and 41, respectively.

Both exonic minisatellites (*MUC2*-MS6 and -MS7) were identified in exon 30 (Figure 1B). *MUC2-*MS6 contained a 24 bp repeat unit (ACCACTCCCAGCCCTCCACCAACC), which encoded an eight amino acid repeat that was threonine (T), serine (S) and proline (P) rich (TTPSPPPT; Figure 1B). *MUC2-*MS7 contained a 69 bp repeat that encoded a 23 amino acid proline/threonine-rich sequence (PTTTPITTTTTVTPTPTPTGTQT; Figure 1B). The primers used for the *MUC2-*MS6 and -MS7 regions were designed against genomic sequence from the UCL homepage. *MUC2-*MS6 could be detected by PCR (Figure 2A–e). We found 5 alleles in 457 controls samples and the number of repeats varied between 40 and 54, with 45 repeats present in the most common allele (93.7%). MS7 (69 bp repeat unit in exon 30) could not be identified by PCR due to its size (>7 kb). However, it was detected in a previous study by Southern blot analysis [9].

In summary, eight novel minisatellites were identified in *MUC2*, six of which were polymorphic and two of which were monomorphic. The high density of minisatellites in this gene may relate to its location near the telomere [17].

### Mendelian inheritance of polymorphic minisatellites in *MUC2*

In order to perform a segregation analysis of minisatellites in *MUC2,* we selected family groups with two and three generations (4 and 3 families, respectively). Blood was collected from the grandparents (GF and GM), parents (F and M) and children (1 to 3) of each family. In controls, six polymorphic minisatellites exhibited heterozygosity that ranged between 0.125-0.977 (Table S1). Figure 2B illustrates the hereditary segregation of six minisatellites during three generations of a family. In seven families, alleles of MS1, MS2, MS3, MS4, MS6 and MS8 could be identified and their transmission traced from parent to child. These results demonstrate that minisatellites in *MUC2* are subject to Mendelian inheritance (i.e., children carried 1 minisatellite allele from each parent). New minisatellite alleles were not observed during this analysis. Thus, the minisatellites in *MUC2* are meiotically stable in seven families and could potentially be used as markers to follow meiotic segregation of *MUC2* alleles.

### Genetic susceptibility to cancer

Since minisatellites are genetically variable, it seems possible that they could play a role in activating *MUC2* during tumorigenesis. This possibility was tested by comparing the distribution and frequency of the polymorphic *MUC2* minisatellite alleles between controls and cancer patients with gastric, colon and rectal cancers.

**Figure 3:**
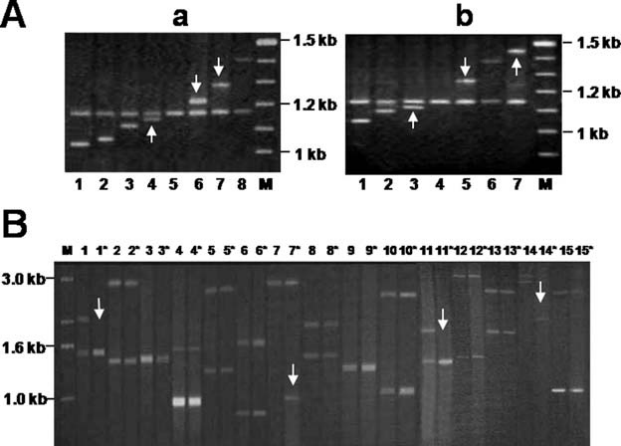
*MUC2* minisatellites in cancer tissues. A. Rare, cancer-specific alleles are identified in *MUC2*-MS6. Comparison of rare cancer-specific alleles of *MUC2*-MS6 between control (Figure 2A-f) and patients with gastric (a), and colon cancer (b). In patients with gastric cancer, four rare cancer-specific alleles were identified (a); and in patients with colon cancer, two rare cancer-specific alleles were identified (b). Haplotype patterns are numbered for *MUC2*-MS6 in gastric (a) and colon cancer (b). Rare cancer-specific alleles are indicated by arrows in (a) and (b). Size markers (M) are given in kb. B. Instability of *MUC2* minisatellites in blood and cancer tissue from patients with gastric tumors. Genomic DNA was analyzed from the blood and gastric cancer tissue of patients. The sizes of minisatellites were analyzed by PCR. Results are shown for *MUC2*-MS2. Gastric cancer tissue samples are indicated by asterisks and M indicates the size marker. Rearrangements in cancer tissues are indicated by arrows.

For further analysis, each *MUC2* minisatellite allele was grouped into two sets (common and rare alleles), according to their frequency in the control population. The expected frequency for rare alleles was considered ≤1% in this study. Table 1 summarizes the frequency of rare and common alleles for the *MUC2-*MS6 among cancer cases and controls. In patients with gastric cancer, the rates of rare *MUC2-*MS6 alleles were 3.3%, compared to 1.3% in cancer-free controls. Analysis of these data revealed a statistically significant association between rare alleles and odds of cancer: *MUC2-*MS6 for gastric cancer (OR, 2.56; 95% CI, 1.31–5.96; *p* = 0.0047). Furthermore, rare cancer-specific *MUC2-*MS6 alleles were found in patients with gastric (3 alleles: 44, 47 and 50 copies; Figure 3A–a) and colon cancers (3 alleles: 44, 50 and 57 copies; Figure 3A–b). We focused on the association between rare alleles and gastric cancer. Rare alleles could be divided into short (40, 43 and 44) and long (47, 50 and 54), according to their tandem repeat lengths. Interestingly, short rare alleles were associated with a relative probability of 5.6 (CI: 1.93–16.42; *p = *0.00036) for gastric cancer (Table 2). Analysis of the number of rare alleles found in individual cancer cases and healthy controls revealed that having at least one rare allele (C/R or R/R) in *MUC2-*MS6 (cases : controls = 6.4%∶2.6%) was associated with a relative gastric cancer odds of 2.52 (CI: 1.27–5.01; *p* = 0.0063) (Table 3). Two rare alleles (R/R) were only detected in one patient with gastric cancer.

**Table 1:** Frequency of rare *MUC2*–MS6 alleles and risk of cancer.

MS6	Analyzed alleles	Common alleles			Rare alleles								OR (95% CI)	*p*	χ^2^-value**
		41	45	Total (%)	40	43	44	47	50	54	57	Total (%)			
**Control**	914	46	856	902 (98.7)	1	3				8		12 (1.3)	1.00 (Reference)		
**Gastric cancer**	**910**	**52**	**828**	**880 (96.7)**	**1**	**17**	**4**	**2**	**3**	**3**		**30 (3.3)**	**2.56 (1.31–5.04)**	**0.0047** *	**7.12**
**GC-Group I**	470	27	426	453 (96.4)	1	10	2	2	1	1		17 (3.6)	2.82 (1.34–5.96)	0.0046*	6.95
**GC-Group II**	440	25	402	427 (97.0)		7	2		2	2		13 (3.0)	2.29 (1.04–5.05)	0.036*	3.56
**Colon cancer**	384	9	366	375 (97.7)		2	2		1	2	2	9 (2.3)	1.93 (0.93–4.04)	0.07	1.22
**Rectal cancer**	542	27	508	535 (98.7)		4	2			1		7 (1.3)	1.17 (0.53–2.59)	0.71	0.04

GC-Group I and II represent two different groups with gastric cancer. They were obtained from two different hospital groups in two different cities (Dong-A University [I] and Chungbuk University [II]).

* Statistically significant (*p*<0.05).

** Pearson's Chi-squared test with simulated p-value (based on 10,000 replicates).

**Table 2:** Frequency of *MUC2*–MS6 alleles and risk of gastric cancer according to short/long tandem **repeat length.**

MS6	Analyzedalleles	Common alleles			Short rare alleles				Long rare alleles			
		41	45	Total	40	43	44	Total	47	50	54	Total
**Control**	914	46	856	902 (98.7%)	1	3	0	4 (0.4%)	0	0	8	8 (0.9%)
**Gastric cancer**	910	52	828	880 (96.7%)	1	17	4	22 (2.4%)	2	3	3	8 (0.9%)
**OR (95% CI)**		1.0 (Reference)			**5.6 (1.93–16.42)**				1.03 (0.38–2.74)			
***p***					**0.00036** *				0.96			
**χ** **^2^** **-value** **					**12.42**				0.006			

Samples were obtained from two different hospital groups in two different cities (Dong-A University [I] and Chungbuk University [II]).

* Statistically significant (*p*<0.05).

** Pearson's Chi-squared test with simulated p-value (based on 10,000 replicates).

**Table 3:** Frequency of at least one rare *MUC2*–MS6 allele in cancer cases and the risk of cancer.

MS6	Total case (allele)***	C/C	C/R+R/R		OR (95% CI)	*p*	χ^2^-value**
**Control (%)**	457 (914)	445 (97.4%)	12	12 (2.6%)	1.00 (Reference)		
**Gastric cancer (%)**	**455 (910)**	**426 (93.6%)**	**28+1** ****	**29 (6.4%)**	**2.52 (1.27–5.01)**	**0.0063** *	**6.01**
**GC-Group I**	235 (470)	219 (93.2%)	15+1****	16 (6.8%)	2.71 (1.26–5.83)	0.0081*	5.39
**GC-Group II**	220 (440)	207 (94.0%)	13	13(5.9%)	2.33 (1.04–5.19)	0.034*	3.30
**Colon cancer (%)**	192 (384)	183 (95.3%)	9	9 (4.7%)	1.96 (0.93–4.13)	0.07	1.13
**Rectal cancer (%)**	271 (542)	264 (97.4%)	7	7 (2.6%)	1.17 (0.52–2.61)	0.70	0.04

GC-Group I and II represent two different groups with gastric cancer. They were obtained from two different hospital groups in two different cities (Dong-A University [I] and Chungbuk University [II]).

* Statistically significant (*p*<0.05).

** Pearson's Chi-squared test with simulated p-value (based on 10,000 replicates).

*** One case had two alleles: C/C, two common alleles; C/R, one rare and one common allele; R/R, two rare alleles

[****One gastric cancer case had two rare alleles].

Supplemental Table S2 summarizes the frequency of rare *MUC2-*MS6 alleles according to age at diagnosis. In the control group, we found that there was no difference in the frequencies of short rare alleles between younger (<50 years) and older individuals (≥50 years) (Table S2-1). In comparison to older patients (≥50 years), younger individuals (<50 years) with gastric cancer had an increased odds (OR = 1.80, CI: 0.71–4.56) of having short rare *MUC2-*MS6 alleles but this was not statistically significant (Table S2-2). Specifically, a comparison of the normal controls and the cancer cases showed the following differences in the association ratios between gastric and short rare *MUC2-*MS6 alleles in younger- and older-patients: younger, 7.79 (CI: 0.94–64.53; *p = *0.026) *vs*. older 5.15 (CI: 1.48–17.93; *p = *0.004) (Table S2–3). The frequency of short rare alleles was higher in younger cases (<50 years) than in older cases (≥50 years). These results suggest that rare *MUC2*-MS6 alleles may be genetically associated with cancer. This is the first report in which minisatellites of the complete *MUC2* region have been characterized in detail, and our observations suggest that *MUC2* minisatellite loci may function as indicators of cancer risk.

We used additional clinicopathological information obtained from the 2004 Nationwide Gastric Cancer Report in Korea [28] and from between 2002 and 2005 at Dong-A University (Table S3). We examined the association between short rare alleles and cancer according to differentiation (for tubular adenocarcinoma), Lauren's classification, N stages and TNM stages (Table S3). We analyzed the gastric tumors according to their classification, and then estimated the frequency of each stage in the total gastric cancer group and in the short rare allele group by Pearson's chi-squared test (Table S3). Tubular adenocarcinomas represented *ca*. 90% of the gastric carcinomas. The gastric cancers were divided into the following differentiation groups: well (35.3%), moderate (28.0%) and poor (36.7%). The frequencies of short rare alleles in these classes were 15.7%, 21.1% and 63.2%, respectively. Thus, the frequency of short rare allele cases in the ‘poor’ differentiation group was significantly higher than in the total gastric adenoma group (*p*<0.0004). These results indicate an increased frequency of short rare alleles in ‘poor’ differentiated cases and a reduced frequency in well differentiated carcinomas. Moreover, the frequency of diffused-type gastric cancer (according to Lauren's classification) was higher in short rare allele group than in the total cancer group (*p*<0.038). However, we found a similar proportion of TNM stage and N stages between the short rare alleles group and the 455 gastric cancer group.

### Analysis of minisatellite instability in cancer tissues

*MUC2* contains a high density of minisatellites that may play a role in its chromosomal instability. This idea was examined by comparing the polymorphic alleles of hypervariable *MUC2*-MS2 minisatellites (heterozygosity>90%) in the blood and cancer tissues from 28 patients with gastric cancer. These cancer tissues were not included in determination of rare alleles for each minisatellite because of their small sample size. In DNA obtained from both blood and cancer tissue of patients with gastric cancer, there were four cases of small deletions or loss of heterozygosity (LOH) in *MUC2*-MS2 in DNA obtained from cancer tissues (Figure 3B). Among the 28 gastric cancer patients, the frequency of rearrangement was 14.3% in *MUC2*-MS2.

## Discussion

Mucins are the major components of mucus, which covers the delicate epithelial surfaces of the intestines, airways and reproductive tracts. All *MUC* genes contain a centrally-located region of sequence that encodes tandem repeats and most mucin genes exhibit a high degree of genetically-determined polymorphism, due to variation in the number of tandem repeats in the TR domain [1]. Sequence analysis of the entire *MUC2* gene allowed the identification of nine tandem repeats. Eight of these are novel minisatellites that can be detected by PCR. Following analysis of the GenBank database using the programs BLASTN, we found no significant similarity between the eight novel minisatellites and such regions identified previously. Thus, all the minisatellites examined in this study are unique to *MUC2* and the properties they confer may be related directly to *MUC2* function.

Minisatellites are tandemly-repeated DNA sequences that are dispersed throughout the genome and which are conserved in humans and other mammals. However, the role that minisatellites play remains unclear. A characteristic of these repetitive sequences is their ability to give rise to variants that contain increased or decreased numbers of repeats. Minisatellites in the 3′-region flanking *H-ras* bind the *rel/NF-κB* family of transcription factors, which contribute to the transcriptional activation of *H-ras* [29]. It is possible that polymorphism in a 46 bp VNTR of human interleukin-1α [30] is also relevant to gene function, since this repeat was described as containing three potential transcriptional factor-binding sites. Similarly, the *hTERT* VNTR 2-2 contains CACGT-binding sites for the *MYC* family of oncogenic transcription factors [15] and this gene has at least six alternative splicing sites, one of which (β site) produces mRNA lacking exons 7 and 8 that results in a protein that is catalytically-inactive and which has a C-terminal truncation [31]. In *MUC2*-MS5 and -MS8 (introns 26 and 40, respectively) we also found several canonical CACGT-binding sites for the *MYC* family of oncogenic transcription factors. Furthermore, alternatively-spliced transcript variants of *MUC2* that include variations in intron 30 and exon 30 have been described previously [32]. Therefore, our data suggest that these minisatellites may contain sequences that are involved in two major regulatory mechanisms for *MUC2*: transcriptional control of *MUC2* and alternative splicing of *MUC2* transcripts.

Rare alleles of VNTRs are associated high risk for various types of cancer [5]. VNTR analysis of *MUC1* found an association between short alleles and gastric cancer [7]. It is now apparent that mutations in repetitive sequences can cause human disease, including several disorders that are associated with a dominant mode of inheritance and cancer [33]. *MUC2* has six polymorphic (MS1, MS2, MS3, MS4, MS6 and MS8) minisatellites and their segregation within families indicated that these minisatellites were transmitted through meiosis, following Mendelian inheritance. Therefore, these polymorphisms could be useful as markers for meiotic segregation of *MUC2* minisatellites during study of *MUC2*-related inheritable diseases.

In this study, we examined allelic variation in *MUC2* and suggested that these variations may represent candidates for susceptibility to gastrointestinal cancer. A case-control study was performed using PCR-based methods to score *MUC2* minisatellite alleles in DNA from cancer-free controls and individuals with gastric, colon and rectal cancers. Interestingly, the frequency of short rare alleles was associated with a relative probability of 5.6 (CI: 1.93–16.42; *p = *0.00036) for gastric cancer. These data suggest that the incidence of short rare *MUC2*-MS6 alleles is significantly higher in gastric cancer patients than in cancer-free controls when sex and age are taken into consideration. These results suggest that rare *MUC2*-MS6 alleles may be genetically associated. This is the first report in which minisatellites have been characterized in detail for the complete *MUC2* gene and our observations suggest that the loci of *MUC2* minisatellites may function as indicators of cancer risk in gastric cancers. In an attempt to compare other cancer type and diseases of the respiratory tract, we analyzed lung cancer and asthma (Leem et al., unpublished results). Interestingly, the rare *MUC2-*MS6 alleles may associate with relative high risk of lung cancer, but no with a risk of asthma. However, the numbers of cases are insufficient at the present time to make a significant statistical analysis (Leem et al., unpublished results). As we mentioned above, *MUC2*-MS6 is located within an exon. We speculate that the proposed association between short rare *MUC2*-MS6 variants and gastric cancer may reflect substantial genetic variation in the serine- and threonine-rich TR units of mucin glycoproteins, which could lead to the construction of inappropriate O-glycosylation structures.

We found a similar proportion TNM stage and N stage cancers between the short rare alleles group and the 455 gastric cancer group. This result suggests that there is no relationship between the short rare allele and the appearance of metastasis in regional lymph nodes. Hence, the frequencies of the short rare alleles differed with respect to with respect to the differentiation of tubular adenocarcinomas. Moreover, the frequency of these alleles was higher in the diffused-type of gastric cancer (Lauren's classification) relative to the total cancer group. Poorly-differentiated and diffused-type gastric cancer tissues are associated with a poor prognosis [34]. These results may suggest that gastric cancer cases with such rare alleles have a bad prognosis.

It has been suggested that genomic instability plays an important role in cancer by accelerating the accumulation of genetic changes that are responsible for cancer cell development [35]. At the DNA level, genomic alterations include loss of heterozygosity (LOH), mutation and microsatellite instability. Microsatellites and minisatellites are divided by their length. Minisatellites form clusters ≤20 kb in length, with repeat units ranging between 10 and 100 bp; microsatellite clusters are shorter, usually 50 to 200 bp long, and the repeat unit is usually 10 bp or less [36]. *MUC2* encodes a secreted gel-forming mucin and often, its expression is reduced in colorectal adenocarcinoma, whereas expression is unaltered in mucinous carcinomas, a distinct subtype of colon cancer associated with microsatellite instability [37]. We examined the *MUC2*-MS2 minisatellites in DNA from blood and cancer tissue derived from 28 patients with gastric cancer, and detected four cases with small deletions or loss of heterozygosity (LOH) in *MUC2*-MS2 (Figure 3B); frequency of rearrangement was 14.3%. This frequency is much higher than the minisatellite regions of *H-ras* [38] and *hTERT* [15], [39] that have been associated previously with cancer tissues. Therefore, these results strongly suggest that *MUC2* contains a high density of minisatellites that might be related to its chromosomal instability. This idea warrants further investigation, such as a large scale epidemiological study into the association of minisatellites and cancer risk. Such a study should also provide a helpful reference for understanding the function and complex genomic properties of mucins.

## Methods

### Database searches, analyses and primer construction for tandem repeat regions in *MUC2*

The *MUC2* genomic DNA sequences (37,982 bp) were obtained from the UCL website [21] and from NCBI (AC139749.4). BLAST analysis (NCBI) between *MUC2* mRNA (NM_002457, 1–15,728 bp) and the two genomic sequences indicated major sequence differences in the exon 30 region.

The Tandem Repeats Finder software package [26] was used to analyze minisatellites and other repeated regions. Repeat units between 10 and 100 bp in length that scored >300 in the program algorithm were selected for further analysis. All primers used in this work were designed using Primer3 software [40]. Primers were made by the sequence on UCL site: MS1, F-CCCTTCCCCATCCCCAGCTA & R-GGCACTCACCCCAGCCTCTG; MS2, F-GACCCCACGCTGGTGCTTTC & R-CCCCGAAGTGCACCGAGAAG; MS3, F-GGCCTTTCCTCAGCCCCAGA & R-GGCTGGTGCACCCACCTTGT; MS4, F-TGTTCAGCATCTGCCACAGCAAG & R-TAGCATGCTCTACGGCACCCTCA; MS5, F- TGCATGGACACTGACACGCAAG & R-GCAGGGGCGAGGAGAGGAAG; MS6, F- TGTTGCTGGCCCATGGATAAGTGT & R-AGGGGTTGTCGTTGAGAATGGTGA; MS7, F-CATCACCACCACCACTACGGTGAC & R-CGGAGGATTGGATGTGGTCAACTC; MS8, F-GTAGGCCCCACCGTGTTT & R-AGAAGCTCTGACATGACATCTTGGCC; MS9, F- CCTCTGCTGTGCCCCTTGAGAG & R-ACCTTCCAGGCACCATCTTGCTC.

### Preparation of genomic DNA from peripheral blood lymphocytes and cancer tissues

The controls and cancer cases had a similar proportion of individuals for sex and age (Table 4). The TNM stages and histopathological characteristics were analyzed according to World Health Organization (WHO) and Lauren classification systems (Table 5). To assess the degree of minisatellite polymorphism in *MUC2*, we analyzed unrelated healthy individuals (Table 1, Table 2 and Table 3). In addition, we performed a case-control study in which we compared the DNA from 457 cancer-free controls with those obtained from individuals with gastric (455), colon (192) and rectal (271) cancers (Table 4 and Table 5). In addition, samples were obtained from seven multi-generational family groups. We have validated our results using two different groups with gastric cancer. All samples were obtained from two different hospitals in two different cities (Dong-A University Hospital [#IRB-06-10-02 & IRB-07-10-7; Busan, Korea] and the Chungbuk National University Hospital [#IRB-2006-1; Cheongju, Korea]). For PCR experiments, genomic DNA was isolated from the peripheral leukocytes, which was taken from 400 µL of whole blood using a Blood and Cell Culture DNA Mini Kit (Qiagen, CA).

**Table 4:** Age and sex distribution of cases and controls.

Characteristic	Level	Controls, [N = 457] N (%)	GC Cases, [N = 455] N (%)	CC cases, [N = 192] N (%)	RC cases, [N = 271] N (%)
**Age (y)**	30–49	100 (21.9)	96 (21.1)	35 (18.2)	52 (19.2)
	50–59	150 (32.8)	121 (26.6)	44 (22.9)	72 (26.6)
	60–69	111 (24.3)	152 (33.4)	64 (33.3)	94 (34.7)
	70–79	87 (19.0)	78 (17.1)	40 (20.8)	48 (17.7)
	80+	9 (2.0)	8 (1.8)	9 (4.7)	5 (1.8)
**Sex**	Women	148 (32.4)	158 (34.7)	75 (39.1)	104 (38.4)
	Men	309 (67.6)	297 (65.3)	117 (60.9)	167 (61.6)

GC, Gastric cancer; CC, colon cancer; RC, rectal cancer

**Table 5:** Tumor characteristics in cases with cancer.

		GC cases		CC cases	RC cases
		No. of cases		No. of cases	No. of cases
**WHO Histological Classification**	Papillary	3	Papillary	1	0
	Tubular adenocarcinoma	402	Tubular adenocarcinoma	178	253
	Mucinous	10	Mucinous	5	5
	Signetring	23	Signetring	0	1
	Others	13	Others	4	0
	Unknown	4	Unknown	4	12
	total	455	total	192	271
**TNM Stage**	0	8	0	4	3
	Ia	195	I	18	63
	Ib	76	IIa	83	88
	II	63	IIb	16	11
	IIIa	59	IIIa	5	21
	IIIb	18	IIIb	40	34
	IV	29	IIIc	21	41
			IV	1	2
	unknown	7	unknown	4	8
	total	455	total	192	271

GC, Gastric cancer; CC, colon cancer; RC, rectal cancer

A total of 28 cancerous tissues and their respective non-cancerous tissues were obtained from patients with gastric cancer and were immediately frozen in liquid nitrogen. Gastric cancer and normal cells were laser capture microdissected using a Pix Cell II LCM system and stained by the HistoGen LCM Frozen Section Staining Kit (Arturus, USA). Malignant cells were captured and their genomic DNA was isolated by using the PicoPure DNA extraction kit (Arturus, USA).

### Analysis of minisatellite polymorphism in *MUC2*

We analyzed *MUC2* minisatellite polymorphisms by PCR, using primers designed against the genomic sequence. PCR reactions (40 µL) were performed in reaction mixes containing 100 ng genomic DNA, 10 µM primers, 2.5 U Go Taq Flexi DNA polymerase (Promega, WI), 50 mM KCl, 10 mM Tris-HCl (pH 9.0), 3.0 mM MgCl_2_, 0.2 mM dTTP, dCTP, dGTP and dATP. PCR was performed in a 9700 Thermocycler (Perkin-Elmer, CT) and the general thermocycling conditions were as follows: 2 min initial denaturation at 94°C, followed by 30 cycles of 30 s at 94°C and 1 min kb^−1^ at 68°C, then a final 7 min extension at 72°C. PCR products were separated by gel electrophoresis (1 volt cm^−1^) using either SeaKem LE agarose (1%, *MUC2*-MS1, -MS2; 1.2%, *MUC2*-MS5, -MS7; 2%, *MUC2*-MS4, -MS6, -MS8; Cambrex, ME) or 3% MetaPhor agarose (*MUC2*-MS3; Cambrex) in 1xTAE buffer.

### Statistical Analyses

****In general, the degree of polymorphism increases with the number of alleles and ranges between 0 and 1. To evaluate the probability of two randomly-chosen alleles being different (heterozygosity) at a given locus, the following formula was used:
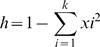
where *xi ^2^* is the allele frequency of the *i*th allele at each minisatellites locus [41].

Regression analyses were performed to determine the odds ratios (ORs) for the association of gastric cancer and short rare *MUC2*-MS6 alleles (or rare *MUC2*-MS6 alleles) between controls and case groups. ORs were estimated using the natural logarithm and its standard error. Where relevant, we used a chi-squared test with one degree of freedom to assess statistical significance. All tests were two-sided, with *p*<0.05 being considered statistically significant. Statistical analyses were performed using MS Excel with CHITEST and R statistical software (v2.5.1, www.r-project.org) with chisq.test for the calculation of chi-squared values.

## Supporting Information

Table S1 Comparison of allelic sizes and frequency of MUC2 minisatellites in controls. Eight novel minisatellites were identified in MUC2 and six minisatellites (MS1, MS2, MS3, MS4, MS6 and MS8) of which were polymorphic. The degree of polymorphism (heterozygosity) is generally increased with numbers of alleles and shown in range 0 to 1.
(0.09 MB DOC)

Table S2 The frequency of short rare MUC2-MS6 alleles according to age at diagnosis. Table S2-1: Frequency of short rare alleles at MUC2-MS6 associated with age in control. Table S2-2: Frequency of short rare alleles at MUC2-MS6 associated with age in gastric cancer cases. Table S2-3: Frequency of short rare MUC2-MS6 alleles and risk of gastric cancer by age.
(0.05 MB DOC)

Table S3 Tumor characteristics in cases with gastric cancer. Analysis of the association between short rare alleles and cancer according to differentiation (for tubular adenocarcinoma), Lauren's classification, N stages and TNM stages. N stage is the N classification of the TNM system and is classified by the appearance of regional lymph nodes: N0; no regional lymph nodes and N1∼N3; metastasis in 1 or more regional lymph nodes. We analyzed the gastric tumors according to their classification, and then estimated the frequency of each stage in the total gastric cancer group and in the short rare allele group by Pearson's chi-squared test.
(0.05 MB DOC)
