# Supplementary material for: Rare Exonic Minisatellite Alleles in MUC2 Influence Susceptibility to Gastric Carcinoma
Source: PLoS One. 2007 Nov 14;2(11):e1163. doi: 10.1371/journal.pone.0001163 (PMC2065792; doi:10.1371/journal.pone.0001163)
Supplement: Table S1 — Comparison of allelic sizes and frequency of MUC2 minisatellites in controls. Eight novel minisatellites were identified in MUC2 and six minisatellites (MS1, MS2, MS3, MS4, MS6 and MS8) of which were polymorphic. The degree of polymorphism (heterozygosity) is generally increased with numbers of alleles and shown in range 0 to 1. (0.09 MB DOC) [file pone.0001163.s001.doc]

**Table S1**

**Table S1.** Comparison of allelic sizes and frequency of *MUC2* minisatellites in controls.

| **MS1 (*h* = 0.499)** | | | | 81 | 2838 | 9 | 0.045 |
| --- | --- | --- | --- | --- | --- | --- | --- |
| Repeats | Size (bp) | N = 800 | Frequency | 83 | 2906 | 4 | 0.02 |
| 12 | 644 | 383 | 0.479 | 84 | 2940 | 2 | 0.01 |
| 15 | 758 | 1 | 0.001 | 85 | 2974 | 7 | 0.035 |
| 16 | 796 | 416 | 0.520 | 86 | 3008 | 12 | 0.06 |
| **MS2 (*h* = 0.977)** | | | | 87 | 3042 | 4 | 0.02 |
| Repeats | Size (bp) | N = 200 | Frequency | 88 | 3076 | 2 | 0.01 |
| 9 | 390 | 2 | 0.01 | 89 | 3110 | 4 | 0.02 |
| 13 | 526 | 2 | 0.01 | 90 | 3144 | 4 | 0.02 |
| 17 | 662 | 2 | 0.01 | 91 | 3178 | 3 | 0.015 |
| 24 | 900 | 1 | 0.005 | 92 | 3212 | 1 | 0.005 |
| 25 | 934 | 3 | 0.015 | 95 | 3314 | 2 | 0.01 |
| 26 | 968 | 2 | 0.01 | 98 | 3416 | 2 | 0.01 |
| 31 | 1138 | 2 | 0.01 | 101 | 3518 | 1 | 0.005 |
| 34 | 1240 | 3 | 0.015 | 102 | 3552 | 1 | 0.005 |
| 35 | 1274 | 5 | 0.025 | 103 | 3586 | 1 | 0.005 |
| 36 | 1308 | 4 | 0.02 | 105 | 3654 | 9 | 0.045 |
| 37 | 1342 | 4 | 0.02 | 106 | 3688 | 3 | 0.015 |
| 38 | 1376 | 3 | 0.015 | 107 | 3722 | 1 | 0.005 |
| 39 | 1410 | 2 | 0.01 | 108 | 3756 | 6 | 0.03 |
| 41 | 1478 | 1 | 0.005 | 110 | 3824 | 8 | 0.04 |
| 42 | 1512 | 5 | 0.025 | 115 | 3994 | 5 | 0.025 |
| 45 | 1614 | 2 | 0.01 | **MS3 (*h* = 0.650)** | | | |
| 46 | 1648 | 2 | 0.01 | Repeats | Size (bp) | N = 400 | Frequency |
| 47 | 1682 | 1 | 0.005 | 25.5 | 475 | 7 | 0.018 |
| 48 | 1716 | 1 | 0.005 | 26.5 | 487 | 135 | 0.338 |
| 49 | 1750 | 1 | 0.005 | 27.5 | 499 | 82 | 0.205 |
| 50 | 1784 | 2 | 0.01 | 28 | 505 | 176 | 0.440 |
| 51 | 1818 | 4 | 0.02 | **MS4 (*h* = 0.542)** | | | |
| 52 | 1852 | 1 | 0.005 | Repeats | Size (bp) | N = 1636 | Frequency |
| 53 | 1886 | 4 | 0.02 | 1 | 190 | 67 | 0.040 |
| 54 | 1920 | 3 | 0.015 | 3 | 320 | 36 | 0.022 |
| 55 | 1954 | 4 | 0.02 | 4 | 370 | 5 | 0.003 |
| 56 | 1988 | 2 | 0.01 | 5 | 430 | 909 | 0.560 |
| 57 | 2022 | 4 | 0.02 | 6 | 490 | 617 | 0.377 |
| 58 | 2056 | 4 | 0.02 | 10 | 690 | 2 | 0.001 |
| 59 | 2090 | 5 | 0.025 | **MS6 (*h* = 0.120)** | | | |
| 60 | 2124 | 1 | 0.005 | Repeats | Size (bp) | N = 914 | Frequency |
| 61 | 2158 | 2 | 0.01 | 40 | 1055 | 1 | 0.0011 |
| 62 | 2192 | 1 | 0.005 | 41 | 1079 | 46 | 0.0503 |
| 63 | 2226 | 1 | 0.005 | 43 | 1127 | 3 | 0.0033 |
| 64 | 2260 | 2 | 0.01 | 45 | 1175 | 856 | 0.9365 |
| 65 | 2294 | 3 | 0.015 | 54 | 1391 | 8 | 0.0088 |
| 68 | 2396 | 3 | 0.015 | **MS8 (*h* = 0.585)** | | | |
| 71 | 2498 | 2 | 0.01 | Repeats | Size (bp) | N = 2458 | Frequency |
| 74 | 2600 | 2 | 0.01 | 6 | 833 | 595 | 0.2421 |
| 77 | 2702 | 5 | 0.025 | 8 | 925 | 470 | 0.1921 |
| 79 | 2770 | 1 | 0.005 | 9 | 971 | 2 | 0.0008 |
| 80 | 2804 | 3 | 0.015 | 10 | 1017 | 1391 | 0.5659 |

***h*,** heterozygosity

**Table S1. Comparison of allelic sizes and frequency of *MUC2* minisatellites in controls.** Eight novel minisatellites were identified in *MUC2* and six minisatellites (MS1, MS2, MS3, MS4, MS6 and MS8) of which were polymorphic. The degree of polymorphism (heterozygosity) is generally increased with numbers of alleles and shown in range 0 to 1.
