# Supplementary material for: Rare Exonic Minisatellite Alleles in MUC2 Influence Susceptibility to Gastric Carcinoma
Source: PLoS One. 2007 Nov 14;2(11):e1163. doi: 10.1371/journal.pone.0001163 (PMC2065792; doi:10.1371/journal.pone.0001163)
Supplement: Table S2 — The frequency of short rare MUC2-MS6 alleles according to age at diagnosis. Table S2-1: Frequency of short rare alleles at MUC2-MS6 associated with age in control. Table S2-2: Frequency of short rare alleles at MUC2-MS6 associated with age in gastric cancer cases. Table S2-3: Frequency of short rare MUC2-MS6 alleles and risk of gastric cancer by age. (0.05 MB DOC) [file pone.0001163.s002.doc]

**Table S2**

**Table S2-1. Frequency of short rare alleles at *MUC2*-MS6 associated with age in control.**

|  | Control | | |
| --- | --- | --- | --- |
| Age at diagnosis | Total cases | Short rare alleles | Total short rare alleles |
| 30-39 | 25 | 0 | 1 (1.0%) |
| 40-49 | 75 | 1 |
| 50-59 | 150 | 2 | 3 (0.8%) |
| 60-69 | 111 | 0 |
| 70-79 | 87 | 1 |
| ≥ 80 | 9 | 0 |
| Total | 457 | 4 | 4 (0.88%) |
| Age at diagnosis | Total cases | Short rare alleles | OR (95% CI); *p* |
| Younger (<50 years) | 100 | 1 (1.0%) | 1.19 (0.12-11.59); *p =* 0.75 |
| Older (≥50 years) | 357 | 3 (0.8%) | Reference |

**Table S2-2. Frequency of short rare alleles at *MUC2*-MS6 associated with age in gastric**

**cancer cases.**

|  | **Gastric cancer cases** | | |
| --- | --- | --- | --- |
| Age at diagnosis | Total cases | Short rare alleles | Total short rare alleles |
| 30-39 | 24 | 3 | 7 (7.3%) |
| 40-49 | 72 | 4 |
| 50-59 | 121 | 5 | 15 (4.2%) |
| 60-69 | 152 | 7 |
| 70-79 | 78 | 3 |
| ≥ 80 | 8 | 0 |
| Total | 455 | 22 | 22 (4.8%) |
| Age at diagnosis | Total cases | Short rare alleles | OR (95% CI); *p* |
| Younger (<50 years) | 96 | 7 (7.3%) | 1.80 (0.71-4.56); *p =* 0.21 |
| Older (≥50 years) | 359 | 15 (4.2%) | Reference |

**Table S2-3. Frequency of short rare *MUC2*-MS6 alleles and risk of gastric cancer by age.**

|  | **Controls** | | **Gastric cancer cases** | | OR (95% CI); *p* |
| --- | --- | --- | --- | --- | --- |
| Age at diagnosis | Total cases | Short rare alleles | Total cases | Short rare alleles | Reference  (Controls of the same age) |
| Younger (<50 years) | 100 | 1 (1.0%) | 96 | 7 (7.3%) | 7.79 (0.94-64.53); *p =* 0.026* |
| Older (≥50 years) | 357 | 3 (0.8%) | 359 | 15 (4.2%) | 5.15 (1.48-17.93); *p =* 0.004* |

* Statistically significant (*p*<0.05)

**Table S2. The frequency of short rare *MUC2-*MS6 alleles according to age at diagnosis.** Table S2-1: Frequency of short rare alleles at *MUC2*-MS6 associated with age in control. Table S2-2: Frequency of short rare alleles at *MUC2*-MS6 associated with age in gastric cancer cases. Table S2-3: Frequency of short rare *MUC2*-MS6 alleles and risk of gastric cancer by age.
