# Supplementary material for: Rare Exonic Minisatellite Alleles in MUC2 Influence Susceptibility to Gastric Carcinoma
Source: PLoS One. 2007 Nov 14;2(11):e1163. doi: 10.1371/journal.pone.0001163 (PMC2065792; doi:10.1371/journal.pone.0001163)
Supplement: Table S3 — Tumor characteristics in cases with gastric cancer. Analysis of the association between short rare alleles and cancer according to differentiation (for tubular adenocarcinoma), Lauren's classification, N stages and TNM stages. N stage is the N classification of the TNM system and is classified by the appearance of regional lymph nodes: N0; no regional lymph nodes and N1∼N3; metastasis in 1 or more regional lymph nodes. We analyzed the gastric tumors according to their classification, and then estimated the frequency of each stage in the total gastric cancer group and in the short rare allele group by Pearson's chi-squared test. (0.05 MB DOC) [file pone.0001163.s003.doc]

**Table S3**

**Table S3. Tumor characteristics in cases with gastric cancer.**

| **Classification** | **Korea Gastric Cancer Assoc.**  **(2004)*** | **2002~2005 patients in**  **Dong-A Hospital** | **Analyzed**  **total case** | **Short rare alleles** | **χ2-value**** | ***p*** |
| --- | --- | --- | --- | --- | --- | --- |
| Total case | 11293 | 989 | 455 | 22 |  |  |
| Average Ages | 58.0 | 57.7 | 58.9 | 55.7 |  |  |
| Differentiation (Tubular ) | 8329 | 831 | 402 | 19 | df = 2  (degree of freedom) | |
| Well | 1517 (18.2%) | 229 (27.6%) | 155 (35.3%) | 3 (15.7%) | **15.53** | **0.0004** |
| Moderate | 3091 (37.1%) | 237 (28.5%) | 123 (28.0%) | 4 (21.1%) |
| Poorly | 3721 (44.7%) | 365 (43.9%) | 161 (36.7%) | 12(63.2%) |
| Lauren’s classification | 7716 | 898 | 261 | 19 | df = 2 | |
| Intestinal | 3699 (47.9%) | 439 (44.4%) | 143 (54.8%) | 7 (36.8%) | **6.55** | **0.038** |
| Diffuse | 3246 (42.1%) | 324 (32.8%) | 90 (34.5%) | 9 (47.4%) |
| Mixed | 771 (10.0%) | 135 (13.7%) | 28 (10.7%) | 3 (15.8%) |
| N Stage | 10795 | 946 | 429 | 18 | df = 1 | |
| N0 | 6254 (57.9%) | 521 (52.7%) | 278 (64.8%) | 12 (63.2%) | 0.0078 | 0.93 |
| N1~N3 | 4541 (42.1%) | 425 (47.3%) | 151 (35.2%) | 7 (36.8%) |
| TNM stage | 11068 | 988 | 429 | 18 | df = 1 | |
| Stage I~II | 7802 (70. 5%) | 701 (71.0%) | 278 (64.8%) | 12 (63.2%) | 0.0078 | 0.93 |
| Stage III~IV | 3266 (29.5%) | 287 (29.0%) | 151 (35.2%) | 7 (36.8%) |

*Data of Korea Gastric Cancer Assoc. (2004) [28], ** Pearson's Chi-squared test.

N stage is the N classification of the TNM system and is classified by the appearance of regional lymph nodes: N0; no regional lymph nodes and N1~N3; metastasis in 1 or more regional lymph nodes.

**Table S3. Tumor characteristics in cases with gastric cancer.** Analysis of the association between short rare alleles and cancer according to differentiation (for tubular adenocarcinoma), Lauren’s classification, N stages and TNM stages. N stage is the N classification of the TNM system and is classified by the appearance of regional lymph nodes: N0; no regional lymph nodes and N1~N3; metastasis in 1 or more regional lymph nodes. We analyzed the gastric tumors according to their classification, and then estimated the frequency of each stage in the total gastric cancer group and in the short rare allele group by Pearson’s chi-squared test.
